# Quaternary Structure Changes for PrP^Sc^ Predate PrP^C^ Downregulation and Neuronal Death During Progression of Experimental Scrapie Disease

**DOI:** 10.1007/s12035-020-02112-z

**Published:** 2020-09-21

**Authors:** Ghazaleh Eskandari-Sedighi, Leonardo M. Cortez, Jing Yang, Nathalie Daude, Klinton Shmeit, Valerie Sim, David Westaway

**Affiliations:** 1 Department of Biochemistry, University of Alberta, Edmonton, AB Canada; 2 Centre for Prions and Protein Folding Diseases, University of Alberta, 204 Brain and Aging Research Building, Edmonton, AB T6G 2M8 Canada; 3 Department of Medicine, Division of Neurology, University of Alberta, Edmonton, AB Canada

**Keywords:** Prion disease, RML, Neuronal loss, PrP^C^ downregulation, PrP assemblies, Asymmetric flow field-flow fractionation

## Abstract

Prion diseases are fatal neurodegenerative diseases in mammals with the unique characteristics of misfolding and aggregation of the cellular prion protein (PrP^C^) to the scrapie prion (PrP^Sc^). Although neuroinflammation and neuronal loss feature within the disease process, the details of PrP^C^/PrP^Sc^ molecular transition to generate different aggregated species, and the correlation between each species and sequence of cellular events in disease pathogenesis are not fully understood. In this study, using mice inoculated with the RML isolate of mouse-adapted scrapie as a model, we applied asymmetric flow field-flow fractionation to monitor PrP^C^ and PrP^Sc^ particle sizes and we also measured seeding activity and resistance to proteases. For cellular analysis in brain tissue, we measured inflammatory markers and synaptic damage, and used the isotropic fractionator to measure neuronal loss; these techniques were applied at different timepoints in a cross-sectional study of disease progression. Our analyses align with previous reports defining significant decreases in PrP^C^ levels at pre-clinical stages of the disease and demonstrate that these decreases become significant before neuronal loss. We also identified the earliest PrP^Sc^ assemblies at a timepoint equivalent to 40% elapsed time for the disease incubation period; we propose that these assemblies, mostly composed of proteinase K (PK)–sensitive species, play an important role in triggering disease pathogenesis. Lastly, we show that the PK-resistant assemblies of PrP^Sc^ that appear at timepoints close to the terminal stage have similar biophysical characteristics, and hence that preparative use of PK-digestion selects for this specific subpopulation. In sum, our data argue that qualitative, as well as quantitative, changes in PrP conformers occur at the midpoint of subclinical phase; these changes affect quaternary structure and may occur at the threshold where adaptive responses become inadequate to deal with pathogenic processes.

**Electronic supplementary material:**

The online version of this article (10.1007/s12035-020-02112-z) contains supplementary material, which is available to authorized users.

## Introduction

Prion diseases are fatal neurodegenerative diseases in humans and animals with the common feature of misfolding and aggregation of the cellular prion glycoprotein (PrP^C^) to protease-resistant “Scrapie” prion protein (PrP^Sc^) [1–3]. The pathogenesis of prion diseases is associated with a progressive accumulation of PrP^Sc^ molecules. Interestingly, prion diseases with different incubation periods share a relatively long sub-clinical stage during which levels of infectious titer do not grow exponentially, but instead reach a maximum [4]. Although the molecular mechanism of this plateau effect, as well as the process through which the subsequent exit to overt clinical disease occurs, is debated, previous work suggests that a decrease in PrP^C^ protein levels at pre-clinical stages of the disease could be of importance in controlling pathogenesis [5, 6].

The notion that different conformational states of PrP^Sc^ exist amongst distinct prion strains is well-established [7–9]. Also, historically, detergent-insoluble protease-resistant PrP^Sc^ species have been defined as the major neurotoxic species and linked directly to disease pathogenesis [10, 11]. However, accumulating experimental evidence obtained from different analytical methods suggests complexities exist even within the confines of one strain with regard to the structural heterogeneity and hints at the presence of different PrP^Sc^ assemblies with distinct biochemical, biophysical, and toxicological characteristics [11–14]. Thus, some studies suggest the presence of a broader spectrum of PrP^Sc^ species with distinct biochemical characteristics and neurotoxicity features at different timepoints during the disease development [10, 14]; the species with conformational characteristics distinct from the material detected at terminal stages of the disease may be responsible for triggering the cascade of events contributing to pathogenesis and neurotoxicity in the brain [6, 15, 16]. Here, we have investigated biochemical transitions of PrP^C^ molecules using fractionation under minimally denaturing conditions, in a mouse model of prion disease (RML) at different timepoints during disease progression. We assessed neural and non-neural cell populations, as well as histological changes of the brain, at the same timepoints to unravel the correlation between PrP^Sc^ species detected at each timepoint and the processes of disease pathogenesis.

## Experimental Procedures

### Animals

FVB/Crl mice were inoculated at 3 to 6 weeks of age by intracerebral (IC) injection of 30 μl of 1% w/v brain homogenate containing either mouse-adapted scrapie (Rocky Mountain Lab “RML” isolate), or normal brain homogenate (NBH). Inoculations into anesthetized animals were made into the left parietal lobe.

All animals were maintained in ventilated racks (Tecniplast, Green Line) and fed irradiated chow (LabDiets, 5053). They were housed with a 12 h/12 h light/dark cycle. Cage environmental enrichment comprised 5 cm diameter plastic tubes and nesting material (“Nestlets”, Ancare Inc.). For the collection of brains for analysis by the isotropic fractionator technique and metabolomic studies, animals were anesthetized by isoflurane inhalation, perfused with 25 mL phosphate saline, the brain was quickly extracted, and one-half brain was stored at 4% phosphate-buffered paraformaldehyde (PFA). The other half brain was snap-frozen and kept at – 80 ^0^C until being processed for metabolic analysis. For immunohistochemistry and western blot analysis, animals were sacrificed by cervical dislocation, and the brain was removed and cut sagittally; one half brain was fixed in Carnoy’s fixation solution (Metha-Carn) [17] for immunohistochemistry analysis and the other half was snap-frozen and kept at – 80 ^0^C until being processed for western blot experiments. All protocols were in accordance with the Canadian Council on Animal Care (CCAC) and were approved by the Animal Care and Use Committee at the University of Alberta (AUP00000357).

### Longitudinal Experimental Design and Sample Collection

The end-stage of the RML prion isolate in mice after i.c. inoculation occurs approximately 150 days post inoculation (DPI). To analyze different aspects of disease progression, we collected samples from both RML- and NBH-inoculated animals at 5 different timepoints: 30, 60, 90, 120, and 150 DPI, the goal being to capture the pathological and molecular events in the brain from the early stages of disease induction (30 DPI), as well as pre-clinical stages (60 and 90 DPI), clinical (120 DPI), and terminal stages (150 DPI). A total of 73 animals (39 females and 34 males) were used in this study. The RML-inoculated cohort consisted of 39 animals (20 female and 19 male), while the NBH cohort consisted of 34 mice (19 female and 15 male). The attack rate for RML-inoculated animals was 100% and the average incubation period was 148 ± 4.1 DPI. The animals were also weighed on a monthly basis. Animals at the terminal stage were euthanized upon presenting overt symptoms including 20% or more weight loss; criteria for scoring disease in RML-inoculated mice are as described previously [18].

### Brain Homogenization

Half brains were weighed and homogenized in ddH_2_O with glass Dounce homogenizers, to make 10% brain homogenates (w/v). Aliquots of solution were prepared and kept at – 80 ^0^C for further analysis.

### Quantification of Brain Cells Using Isotropic Fractionator

The isotropic fractionator method for quantifying neuronal loss in tissue samples was performed as previously described with minor modifications [19, 20]. Briefly, perfused and post-fixed brain samples were mechanically dissociated and homogenized in 10 volumes of a solution of 40 mM sodium citrate and 1% Triton X-100. The homogenates were collected, and the homogenizer was washed at least twice to collect any residual cells. To visualize nuclei and obtain total cell counts, 20 μL of 100 μg/mL stock solution of 4’,6-diamidino-2-phenylindole dihydrochloride (DAPI, Sigma) was added to the cell suspension and the total number of cells was counted (final concentration of DAPI was 0.20 μg/ml) using disposable hemacytometers (inCYTO). Counting was done in a semi-automated manner: pictures of the quadrants were taken using an InCell 2000 analyzer, and a sub-routine software, adjusted to detect fluorescent signals from stained nuclei (InCell Analyzer software, GE-Healthcare), was used for unbiased counting of the cells. All four quadrants in both the upper and lower grids of the hemocytometer were used for counting and the results were averaged. To determine total neuron counts, 1 mL of each cell suspension was removed, washed with PBS, and centrifuged for 10 min at 5000×*g*. Cells were then incubated in anti-NeuN antibody (1:200, rabbit polyclonal; Millipore) for 2 h followed by incubation in a secondary anti-rabbit IgG-Alexa-Fluor 594 for 1 h (1:200; Invitrogen). Cells were then counted in the same semi-automated manner described earlier. The percentage of NeuN-stained nuclei was recorded. Total cell numbers and neuronal numbers were then calculated by multiplying the number of cells/mL by the final volume.

### Immunohistochemistry

Sagittal sections were processed in Carnoy’s fixative solution and paraffin-embedded as above. Hematoxylin and eosin (H and E) staining was done as previously described [21]. For immunodetection, slices were heated to 121 °C in 10 mM citrate buffer and allowed to cool to room temperature. Staining for PrP^Sc^ was then accomplished by treatment with formic acid and 4 M guanidine thiocyanate before an overnight incubation with biotinylated SAF83 (Cayman Chemicals). GFAP (1/1000) immunodetection was accomplished by subsequent incubation in 3% peroxide and overnight incubation with a biotinylated primary antibody cocktail (BD Biosciences; 556330), as well as counterstaining with Mayer’s hematoxylin.

### Western Blotting

Western blotting was performed as described previously [22]. Samples were prepared in loading buffer containing SDS and 2-mercaptoethanol and boiled for 10 min. They were then electrophoresed on 10% bis-tris precast gels (Invitrogen) using an Invitrogen system and transferred to polyvinyl difluoride (PVDF; Millipore) membranes (wet transfer). Blots were then blocked with 5% skim milk in 1xTBS-0.1% Tween 20 for 1 h at room temperature and incubated with primary antibodies at 4 °C overnight (except for blots prepared for detection with Sha31 (Spibio) antibody, where no blocking was done and blots were directly incubated with antibody in 1xTBS-0.5% Tween 20). To assess inflammation markers and synaptic density antibodies against ionized calcium binding adaptor molecule 1-Iba1 (Waco) (used at dilution 1/5000), triggering receptor expressed on myeloid cells 2-TREM2 (Abcam) (used at dilution 1/1000) and synaptophysin (Sigma-Aldrich) (used at dilution 1/1000) were used. To detect PrP, we used the Sha31 antibody (1/30,000 for brain homogenates and 1/5000 for immunoblotting of AF4 fractions). Blots were then incubated with the proper alkaline phosphatase (AP) or horseradish peroxidase (HRP) conjugated secondary antibody and developed by Attophos (Promega; for AP-conjugated antibodies) or ECL (Pierce; for HRP-conjugated antibodies). Fluorescence and chemiluminescence were measured by ImageQuant LAS 4000 (GE Healthcare).

### Proteinase-K Digestion

For proteinase-K (PK) digestion of crude brain homogenates, we used protein/enzyme ratios as previous reports [16]. Briefly, 20 μg of brain homogenate was incubated with 20 μg/mL of PK (New England Biolabs) for 1 h at 37 ^0^C. The reaction was then stopped by addition of Pefabloc (Sigma), and samples were boiled with SDS-sample buffer and electrophoresed on 4–12% NuPAGE gels. For PK digestion of asymmetric flow field-flow fractionation (AF4) fractions, 10 μL of each fraction was incubated with 20 μg/mL of PK in the presence of 20 μg bovine serum albumin (BSA) as the sacrificial substrate to account for the low mass of PrP in eluted fractions, and to assure constant total protein mass in all samples. Reactions were then adjusted to the same volume for all samples and they were incubated with PK for 1 h at 37 ^0^C. The reaction was then stopped by addition of Pefabloc. Samples were then boiled with SDS-sample buffer and separated by SDS-PAGE.

### Asymmetric Flow Field-Flow Fractionation

One hundred microliters of brain homogenates (10% w/v) was solubilized by adding an equal volume of solubilization buffer (50 mM HEPES pH 7.4; 300 mM NaCl; 10 mM EDTA; 4% (w/v) dodecyl-β-D-maltoside (Sigma)) [23], and incubated for 45 min on ice. Sarkosyl (N-lauryl sarcosine; Fluka) was added to a final concentration of 2% and incubated on ice for 30 min. The samples were then centrifuged (20,000×*g*, 10 min) at 4 °C. The supernatants were collected, and 350 μg of total protein was subjected to asymmetric flow field-flow fractionation (AF4) on an AF2000 Postnova system using phosphate-buffered saline solution pH 7.4 containing 0.05% sodium dodecyl sulfate (SDS) as the running buffer. The fractionation channel was 26.5 cm in length and 350 μm in height, constructed with a trapezoidal spacer of maximal width 21 mm at the inlet, and lined with a 10-kDa cut-off polyethersulphone membrane at the accumulation wall. Samples were focused for 4 min and then eluted at a channel flow rate of 0.5 mL/min with constant cross-flow for the first 10 min, decreasing from 1.5 to 0.35 mL/min in the following 15 min, from 3.5 to 0 mL/min in the next 30 min, and running with no cross-flow for the last 10 min. A slot pump was run at 0.3 mL/min to concentrate the samples before they passed through the detectors. Fractions of 0.2 mL were collected. Static and dynamic light scattering measurements were carried out on an in-line DAWN HELEOS II detector (Wyatt Technology) and the data collected and analyzed using ASTRA analysis software (version 6.0). After fractionation, equal volumes of individual fractions were subjected to western blot to quantify the amount of total PrP (Supplementary Fig. 1 and 2). The size distributions of PrP particles were then measured by plotting the acquired PrP signal intensity for each fraction versus the average of thirty hydrodynamic radii (R_H_) values of the particles present in that fraction. The PrP intensity of all AF4 fractions was normalized by the total amount of PrP in the corresponding un-fractionated sample. All unfractionated samples were run in the same SDS-PAGE to compare their relative signal intensity. The size distribution of PrP particles under non-infected conditions (i.e., assemblies deriving from PrP^C^) was determined by fractionation and immunoblotting of NBH-inoculated brains. Two biological replicates were analyzed. Unpaired Student’s *t* test was used for statistical analysis.

### Expression and Purification of Mouse PrP

A codon-optimized synthetic gene corresponding to full length mouse PrP (moPrP23-231; allelic type *Prnp*^a^) was obtained as previously described [24]. *Escherichia coli* BL21 (DE3) cells harboring this plasmid were grown in LB media plus 100 μg/mL ampicillin at 37 °C. Protein expression was induced with addition of 1 mM isopropyl-1-thio-β-d-galactopyranoside. Bacteria cells were then pelleted and resuspended in lysis buffer (8 m urea, 10 mm Tris, 100 mm Na_2_PO_4_, pH 8.0) and sonicated with digital sonifier (Branson 450 digital sonifier) using a microtip at 30% amplitude for 10 cycles of 30 s. Cell debris were removed by centrifugation at 6000×*g* and the supernatant was loaded in a nickel-nitrilotriacetic acid column (Thermo Fisher). Contaminants were removed using 5 column volumes of 8 M urea, 10 mm Tris, 100 mm Na_2_PO_4_, and 50 mm imidazole (pH 6.3). The His-tagged prion protein was eluted using 8 M urea, 10 mm Tris, 100 mm Na_2_PO_4_, and 500 mm imidazole (pH 4.5). The collected sample was then subjected to HPLC for further purification (C4 Vydac preparative column). Aliquots of 8 mL of sample were injected and then eluted using a gradient of acetonitrile (ACN) (20–60%) and 0.1% trifluoroacetic acid in 60 min. The protein eluting at 35–38% ACN was collected, lyophilized, and stored at − 80 °C.

### Real-Time Quaking-Induced Conversion Assay

Recombinant PrP (moPrP) was solubilized in 6 M guanidine hydrochloride (GdnHCl) solution. This was then diluted in real-time quaking-induced conversion (RT-QuIC) buffer (20 mM sodium phosphate pH 7.4; 130 mM NaCl; 10 mM EDTA; 0.002% SDS) to a final protein concentration of 0.2 mg/mL (and residual 0.2 M GdnHCl). Reactions were seeded with 2 μL of 10% solubilized brain homogenate in a final volume of 180 μL/well. The aggregation reactions were carried out in 96-well plates (Costar 3610) sealed with thermal adhesive film (08-408-240; Fisherbrand). We used three different dilutions of terminal-stage (150 DPI) brain homogenates (10^-3^, 10^-5^, and 10^-6^) as positive controls for these reactions and to calibrate sensitivity. NBH-inoculated mice brain homogenate at 10^-3^ dilution was used as a negative control. 10^-3^ dilutions were used to compare seeding activity at different timepoints in prion-infected animals. All test samples were incubated in the presence of 10 mM thioflavin T (ThT) at 42 °C with cycles of 1-min shaking (700 rpm double orbital) and 1-min rest. ThT fluorescence measurements (450+/210 nm excitation and 480+/210 nm emission; bottom read) were recorded every 60 min. There were three technical replicates for each sample per experiment.

### Data Analysis

Histology and western blot data were analyzed using ImageJ software (NIH). For quantification of histology data, we used trainable Weka (Waikato Environment for Knowledge Analysis) segmentation and ImageJ [25]. Briefly, a probability map was created for each histology experiment by a training set that was chosen from 5 control images and prion infected animals (RML). The trained map was then applied to the samples and the measured values were then quantified in ImageJ. Quantification was performed on the images obtained from the sagittal sections of half brains. A minimum of 3 animals were analyzed for each timepoint (per cohort) (for isotropic fractionator *n* = 5). Unpaired Student’s *t* test was used for statistical analysis.

## Results

### Quantitative and Qualitative Changes in Brain Cell Populations During the Course of Infection with the RML Scrapie Prion Isolate

We used a longitudinal experimental design [5, 6, 26], with analyses at sequential 30-day intervals to follow steps in disease pathogenesis. With regard to determining the disease-related endpoint measure of cell loss, we used the isotropic fractionation technique; this is a non-stereological method to quantify different cell populations in the brain based on counting isolated nuclei following detergent lysis of cell membranes and its accuracy has been verified by cross-referencing to other techniques [27–31]; we took advantage of this method to determine the number of cells in the brain at different timepoints in RML inoculated animals, as well as in matched controls.

**Fig. 1:**
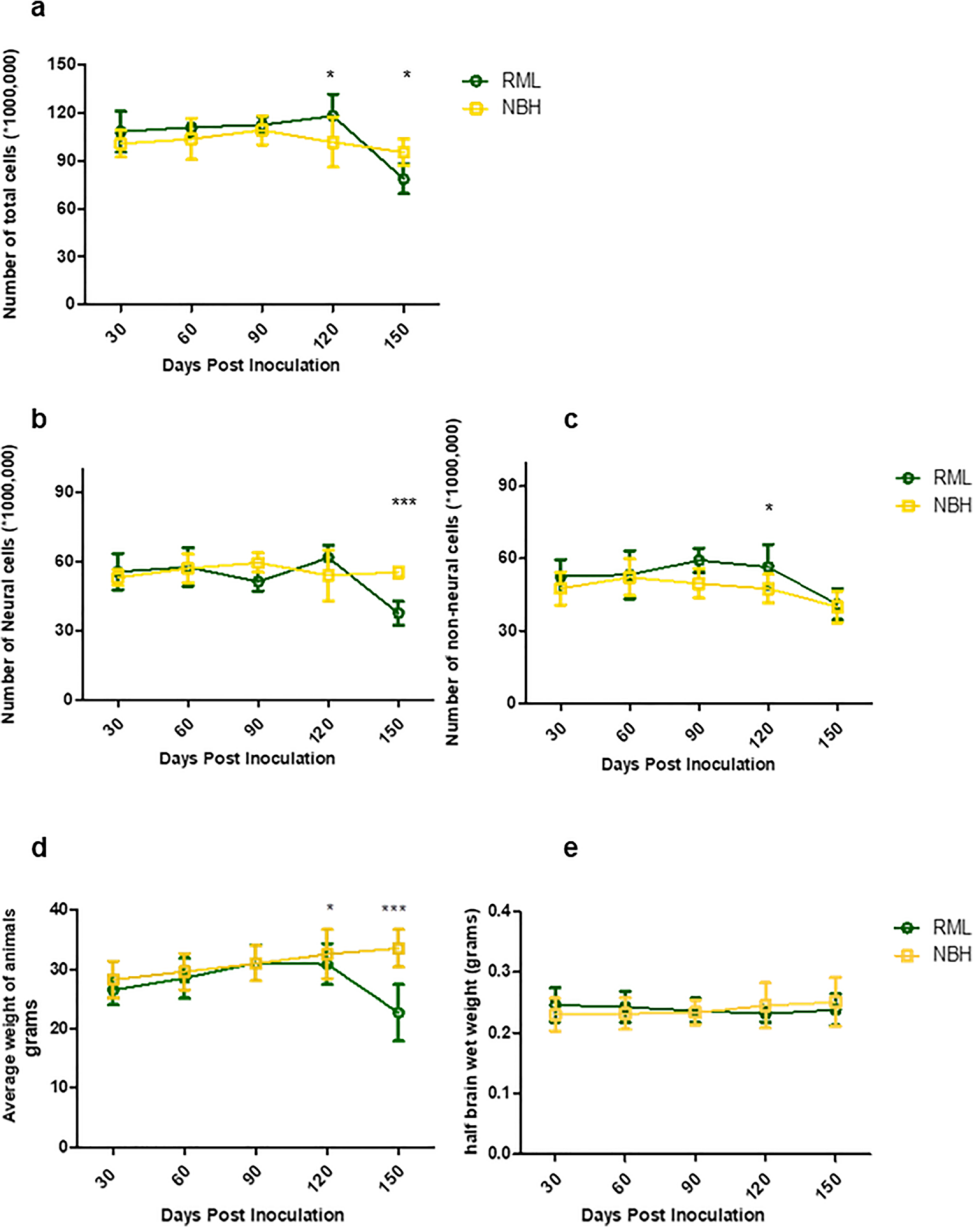
Quantification of brain cell populations by isotropic fractionation. **a** Quantification of total brain cells indicates a significantly higher number of cells at 120 DPI and a significant decrease in the number of cells at 150 DPI in RML-inoculated animals. **b** Quantification of neuronal cells in the brain indicates a significant decrease in the number of neurons at 150 DPI in RML-inoculated animals. **c** Quantification of non-neuronal cell populations suggests a trend towards increase at 90 DPI, and a significantly higher number of cells at 120 DPI in RML-inoculated animals. **d** Monitoring weight changes of NBH and RML-infected animals reveals a significant decrease in animal weight at 120 and 150 DPI in RML-inoculated mice. **e** No significant difference was detected in brain wet weight between NBH and RML inoculated animals. Data obtained from 5 animals were averaged per group in each timepoint. Error bars represent SD. (*p* values * ≤ 0.05, ** ≤ 0.01, and *** ≤ 0.001)

Assessments of neural and non-neural cell populations at different timepoints indicated a significant increase in total brain cells (i.e., total number of nuclei as stained with DAPI) at 120 days post-inoculation (DPI), followed by a significant decrease at 150 DPI (Fig. 1a). Analyses of the NeuN antibody-positive population of nuclei revealed significant neuronal loss at the terminal stage of the disease (150 DPI). In contrast, the non-neural cell population tended to an increase, starting from 90 DPI and reaching significance at 120 DPI (Fig. 1b, c). In parallel to these measurements, we observed a significant decrease in body weight of the RML-infected mice; however, the wet weight of brains showed no significant changes in any of the cohorts thus excluding this parameter as a confound for data interpretation (Fig. 1d, e). Due to the current lack of lineage-specific nuclear markers for any of the non-neuronal cell populations in the brain (i.e., analogous to the use of NeuN for identifying nuclei derived from neurons), we could not distinguish individual cell lineages amongst the non-neuronal cell cohort. However, prior reports indicate microgliosis and astrocytosis initiating at pre-clinical stages of prion diseases [32, 33] and the results here from the isotropic fractionator analyses are potentially compatible with these data.

### Timing of Pathological Changes in the Brain

**Fig. 2:**
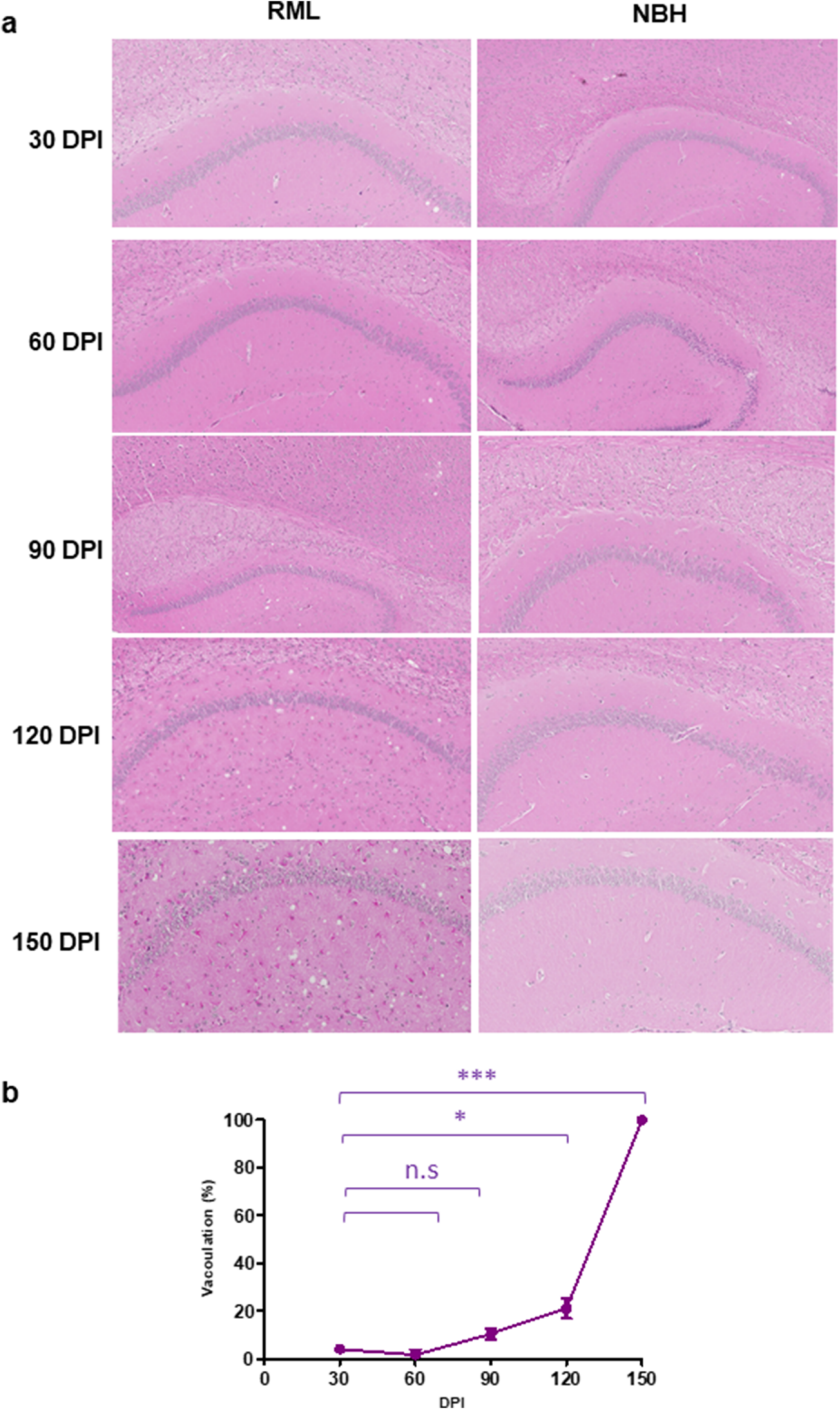
H&E staining of brain sections from RML and NBH inoculated animals at different timepoints during disease progression. **a** A section of the brain consisting of the hippocampus and part of the cerebral cortex are presented. Vacuoles are first noticeable at 90 DPI. **b** Quantification of H&E staining on half-brain sagittal sections by Weka image segmentation and ImageJ. The values obtained at terminal-stage were adjusted to 1 and the rest of the timepoints were normalized relative to that. * indicates *p* value ≤ 0.05 and *** indicates *p* value ≤ 0.001. n.s indicates not significant

**Fig. 3:**
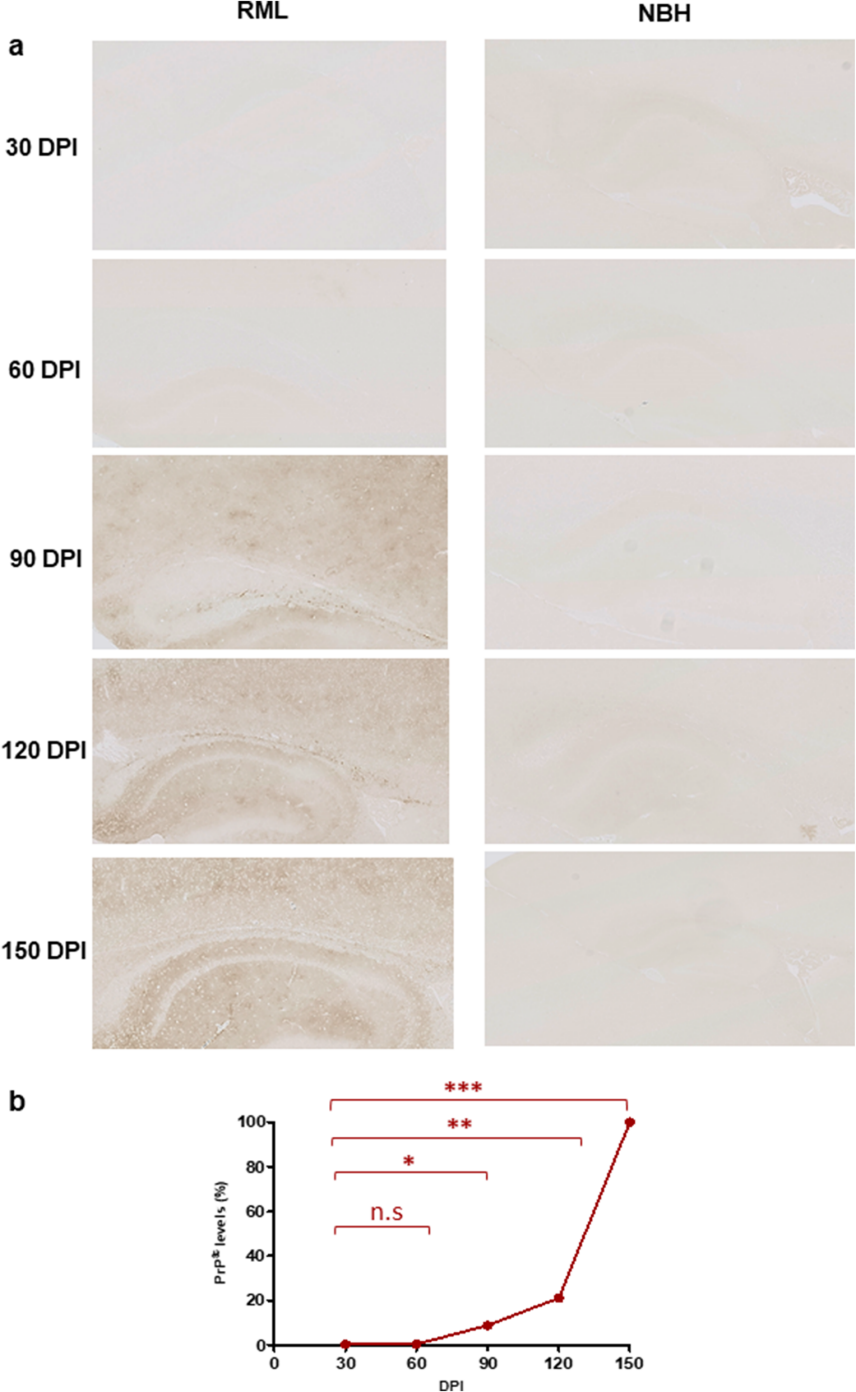
PrP^Sc^ immunostaining of brain sections from RML and NBH inoculated animals at different timepoints. **a** A section of the brain consisting of the hippocampus and part of the cerebral cortex are presented. PK-resistant PrP is first noticeable at 90 DPI. **b** Quantification of PrP immunostaining on half-brain sagittal sections by ImageJ. The values obtained at terminal-stage were adjusted to 1 and the rest of the timepoints were normalized relative to that. * indicates *p* value ≤ 0.05, ** indicates *p* value ≤ 0.01, and *** indicates *p* value ≤ 0.001. n.s indicates not significant

**Fig. 4:**
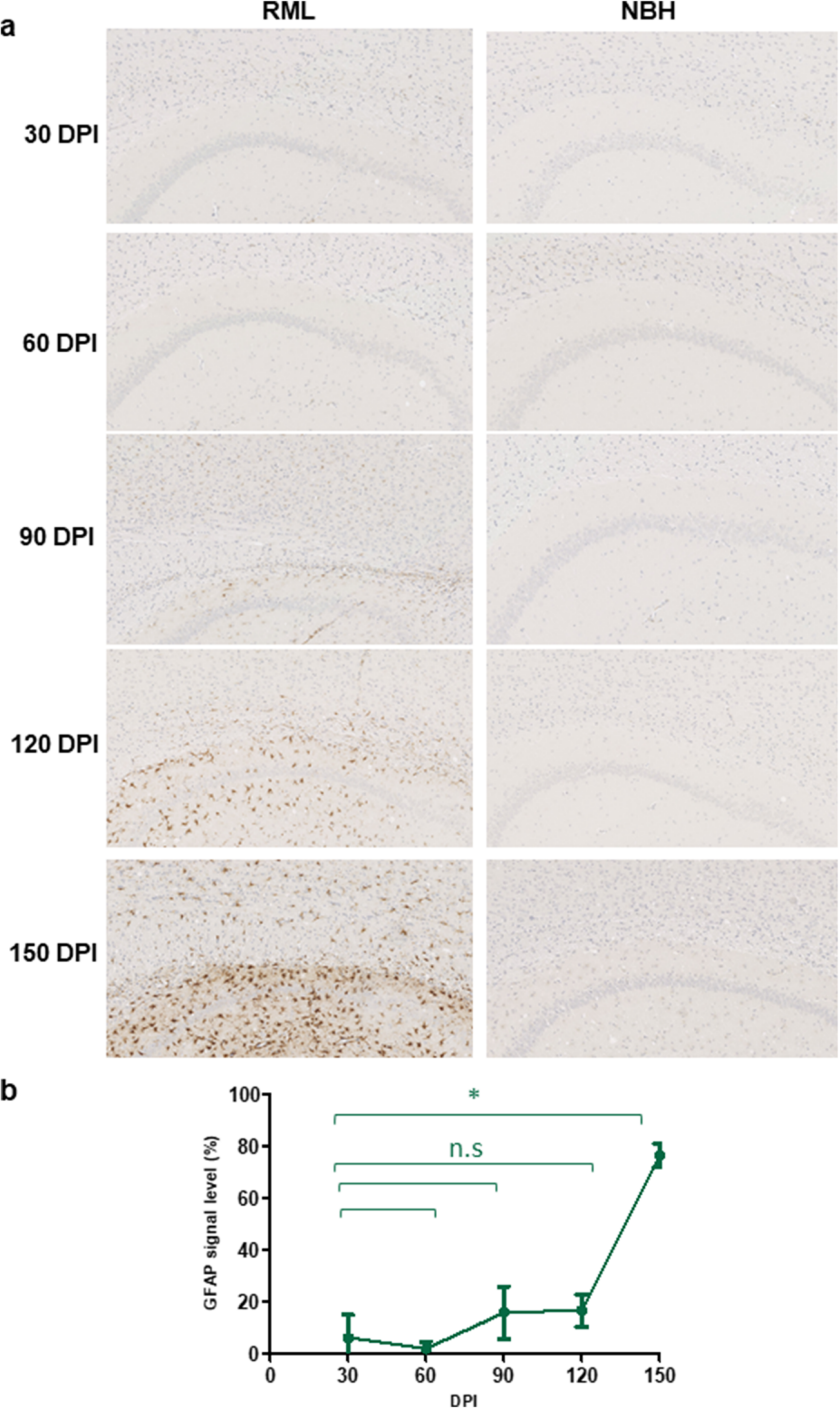
GFAP immunostaining of brain slices from RML and NBH inoculated animals. **a** A section of the brain consisting of the hippocampus and part of the cerebral cortex are presented. Increased GFAP immunoreactivity is first noticeable at 90 DPI. **b** Quantification of GFAP immunostaining on half-brain sagittal sections by ImageJ. * indicates *p* value ≤ 0.05

**Fig. 5:**
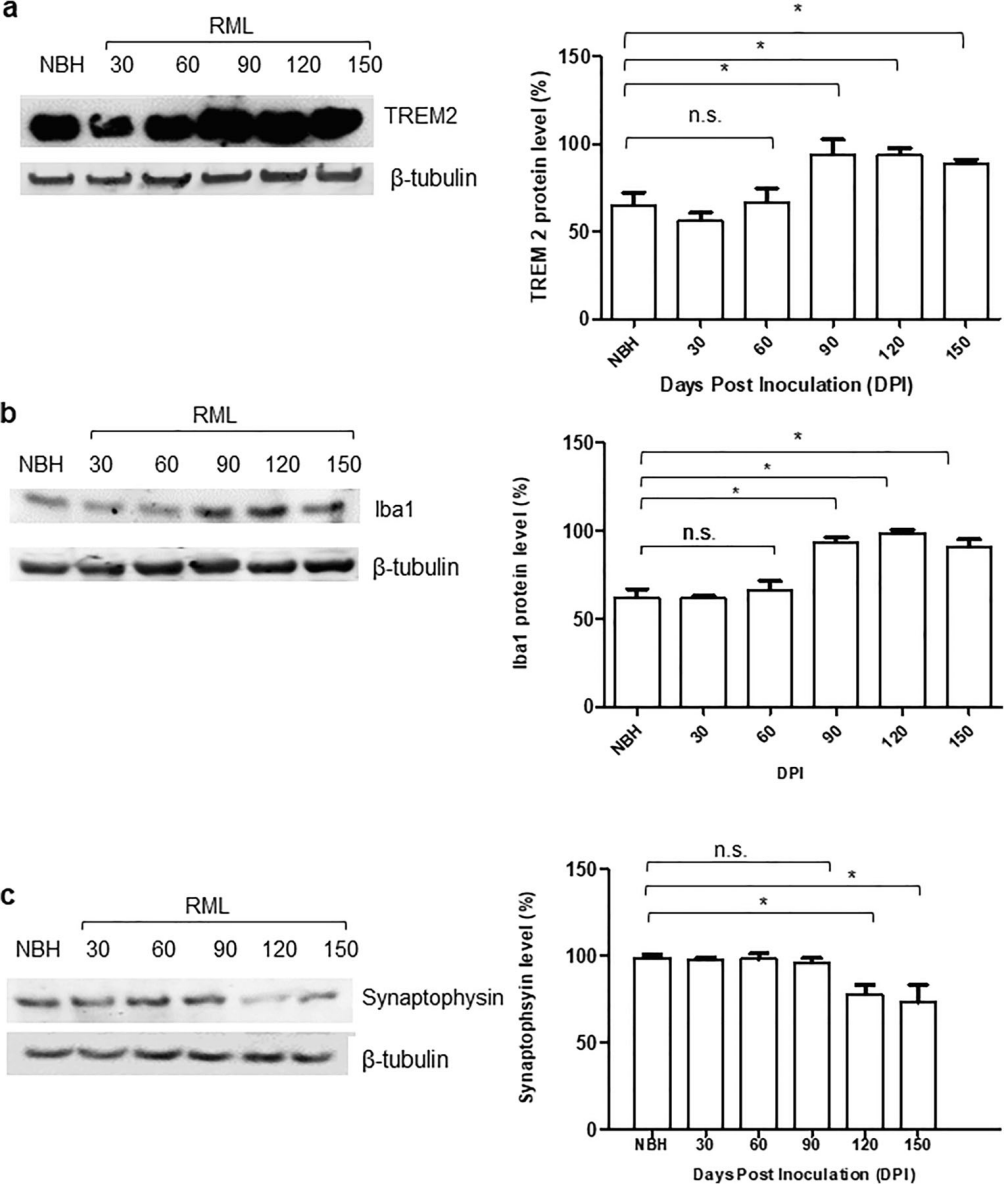
Assessment of inflammatory and synaptic health biomarkers at different timepoints during disease progression. **a** Immunoblotting of TREM2 protein in solubilized brain homogenate of RML-inoculated animals. Antibody (anti-TREM2 1/5000). **b** Immunoblotting of Iba1 protein in solubilized brain homogenate of RML-inoculated animals. Antibody (anti-Iba1 1/5000). **c** Immunoblotting of synaptophysin protein in solubilized brain homogenate of RML-inoculated animals. A 150-DPI sample from an NBH-inoculated animal was used as a control for panels **a**–**c**. Antibody (anti-synaptophysin 1/2000). Quantification of the blots was done by ImageJ. Data represents an average of 3 repeats. Error bars represent SD. * indicates *p* value ≤ 0.05. n.s indicates not significant

**Fig. 6:**
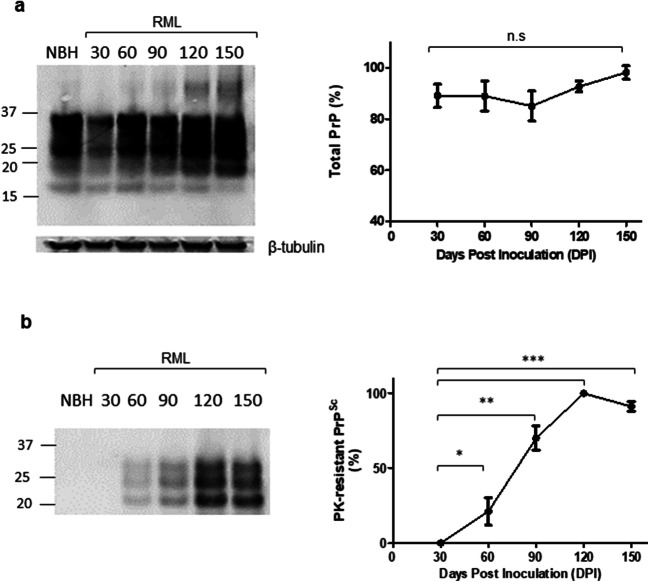
Total and PK-resistant PrP in solubilized brain homogenates. **a** Immunoblotting of total PrP in solubilized brain homogenate of RML-inoculated animals. A NBH-inoculated brain homogenate at 150 DPI is used as the control. **b** Immunoblotting of PK-resistant PrP^Sc^ in solubilized brain homogenate of RML-inoculated animals at different timepoints during disease progression. A NBH-inoculated brain homogenate at 150 DPI was used as a control as per Fig. 5. Antibody Sha 31 (1/30,000). Quantification of the blots was done by ImageJ. Data represents an average of 3 repeats. * indicates *p* value ≤ 0.05, ** indicates *p* value ≤ 0.01, and *** indicates *p* value ≤ 0.001. n.s indicates not significant

To understand the correlation between the molecular transition of PrP and disease progression, we monitored the development of clinical signs and assessed changes to the brain tissue by histology (Figs. 2, 3, and 4). We analyzed the vacuolation (by H&E staining), PK-resistant PrP accumulation, and the presence of glial fibrillary acidic protein (GFAP) as a marker of astrocyte activation at different timepoints during disease progression. Quantification of half brain sections (performed on 3 animals per group by Weka segmentation) indicated the presence of pathological changes in the brain from 90 DPI onwards. We also assessed neuroinflammation, as well as synaptic health, by quantifying protein makers of these events at different timepoints (*n* = 3). For Iba1 and TREM2, which are both exclusively expressed by microglia in the brain [34, 35], significant increases in immunoreactivity were as early as 90 DPI (Fig. 5a, b). The synaptic integrity marker synaptophysin showed significant decrease starting at 120 DPI (Fig. 5c). Clinical symptoms in scrapie-infected mice can be subtle, with some deaths occurring in their absence [36, 37], and in the case of the RML prion isolate with an incubation period measured here at 148 ± 4.1 DPI, symptomatic changes in infected animals may only be scored consistently after 120 DPI. The cohort of RML-inoculated animals manifested significant weight loss at 120 DPI and by 150 DPI (Fig. 1d). We also investigated the total PrP and PK-resistant PrP content in the brains of RML-inoculated animals at different timepoints (Fig. 6). We detected an increase in total PrP levels at 120 and 150 DPI (statistically not significant) (Fig. 6a) and a significant increase in PK-resistant PrP^Sc^ levels from 60 DPI onwards (Fig. 6b). Thus, the combination of histology and immunoblotting experiments point to discernible changes in the brain of RML-inoculated animals at 90 DPI and thereafter (Figs. 2, 3, 4, 5, and 6), with 90 DPI considered to fall within the preclinical phase of disease.

### Molecular Transitions of PrP: Decrease in PrP^C^ Levels and Presence of High-Molecular Weight Species at Early Stages of the Disease

To understand the evolution of PrP forms during disease progression, we used asymmetric flow field-flow fractionation (AF4), a flow-based separation method for isolation of macromolecules based on hydrodynamic radius. An optimized solubilization condition using 2% dodecyl-β-D-maltoside and 2% sarkosyl was used as an initial step for processing brain homogenates and has been variously advocated by others as preserving a natural disposition of different types of PrP^Sc^ assemblies, including less soluble aggregates [23, 38–41].

**Fig. 7:**
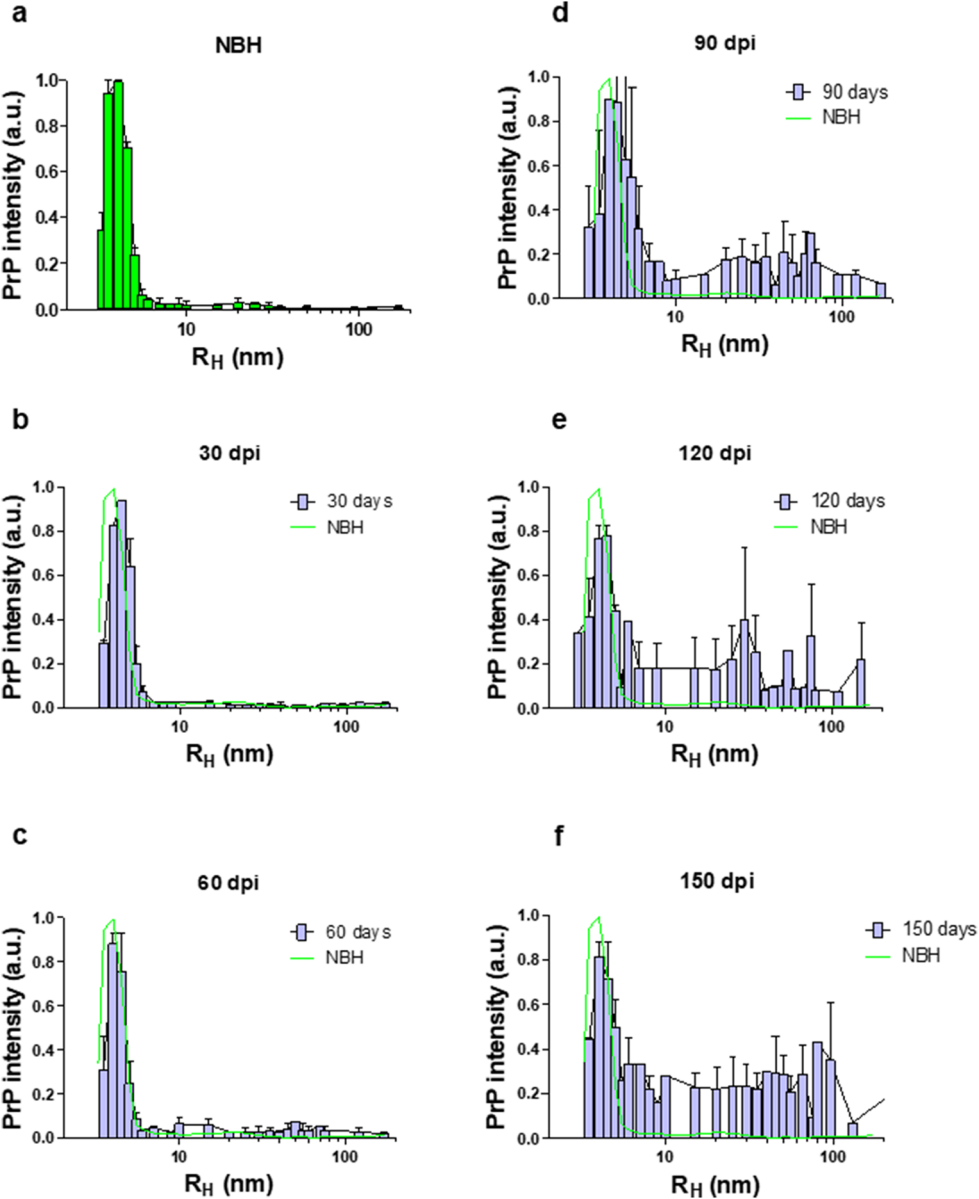
AF4 analysis of solubilized brain homogenate of RML-infected animals at different timepoints during disease progression. The *Y*-axis represents the intensity of total PrP in arbitrary units (see supplementary Fig. 1). **a** A representative graph from averaged fractionated brain of NBH-inoculated animals at 150 DPI, **b** fractionated brain of RML-inoculated mice sacrificed at 30 DPI, **c** fractionated brain of RML-inoculated mice sacrificed at 60 DPI, **d** fractionated brain of RML-inoculated mice sacrificed at 90 DPI, **e** fractionated brain of RML-inoculated mice sacrificed at 120 DPI, and **f** fractionated brain of RML-inoculated mice sacrificed at terminal stage (150 DPI). Data represent an average from 2 biological replicates (i.e., 2 animals). Error bars represent SD

All PrP^C^ in NBH-inoculated control samples eluted within the first 16 fractions, with the maximum PrP signal intensity seen for particles with R_H_ of ~ 4 nm, and almost no signal in particles larger than 6 nm (Fig. 7a). Secondly, regarding the high-molecular weight assemblies of PrP, a comparison of PrP size range in NBH-inoculated and RML-inoculated animals suggests that assemblies of PrP with R_H_ values within the range of 10–100 nm are mostly absent in healthy conditions (Fig. 7a–f and Supplementary Fig. 1). This range of assemblies in RML-infected animals would consist of at least 23 PrP monomers (Table 1).

**Table 1:** Full-length PrP molecules per oligomer as a function of R_H_. PrP particle R_H_ values and the correlated number of PrP monomers present in each particle were calculated by the Zetasizer Software 7.11. These calculations were done based on the molecular weight of PrP^C^ which is 32 kDa. The particle shape is presumed as spherical for all these calculations. The measured values for very high-molecular weight assemblies (presented with *) are not as reliable since these particles probably consist of protofibrils and fibrils

R_H_ (nm ± SD)	Number of PrP monomers in the complex (full length)
2.6 (± 0.05)	(PrP)_1_, monomer
3.5 (± 0.52)	(PrP)_2_, dimer
7 (± 0.62)	(PrP)_10_, decamer
10 (± 0.85)	(PrP)_23_, mer
20 (± 1.55)	(PrP)_115_, mer
40 (± 6.16)	* (PrP)_550_
70 (± 10.63)	* (PrP)_2000_

**Fig. 8:**
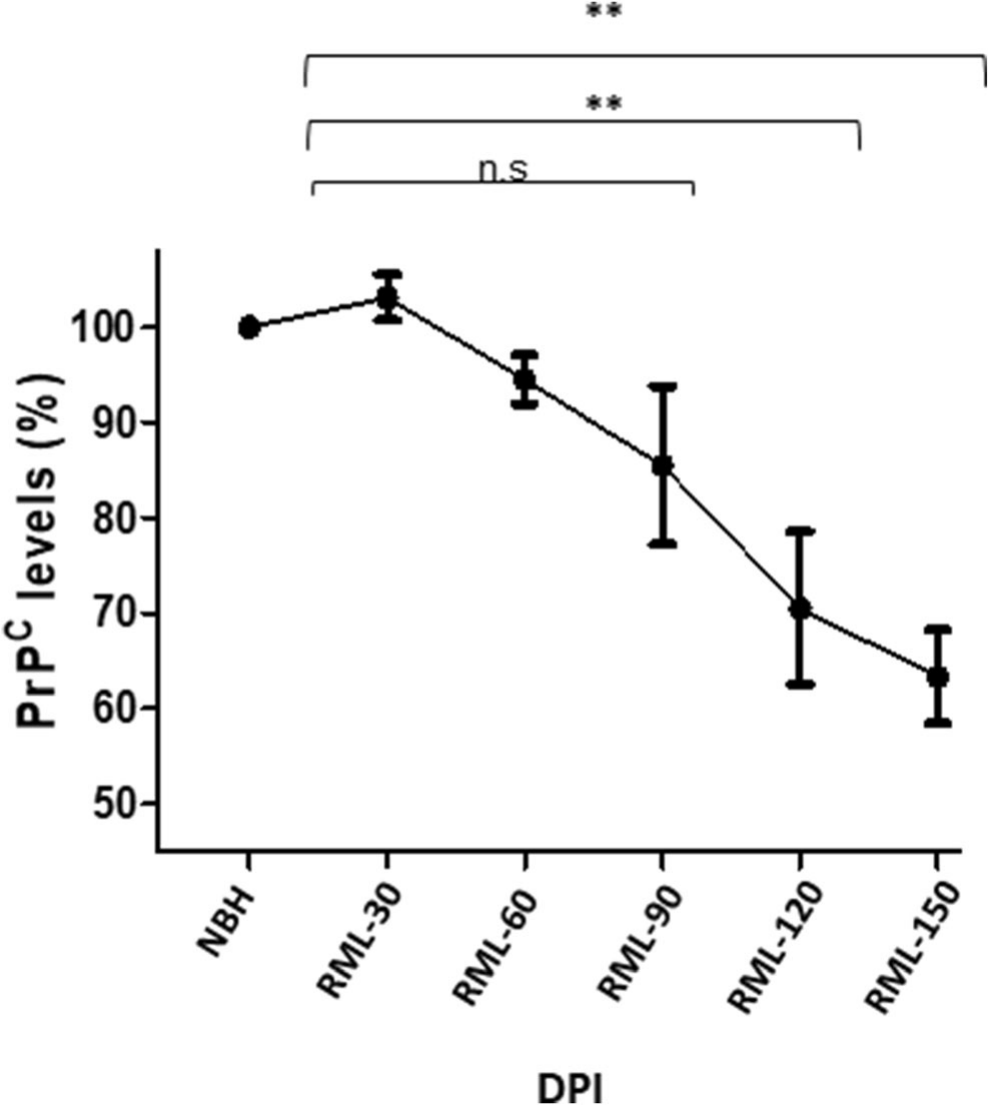
PrP^C^ levels in brain homogenate of RML-inoculated animals at different timepoints. Protein levels were quantified by integrating the values from fractions corresponding to PrP^C^ obtained from fractionation of brains from NBH-inoculated mice (green trace, see Fig. 7b–f) and expressing the integrated values from infected animals as a percentage of this reference value. There is a trend towards decrease at 90 DPI, and a significant reduction at 120 and 150 DPI (29% and 38% respectively). Biological replicates are as per Fig. 7. Error bars represent SD. ** indicates *p* value ≤ 0.01. n.s indicates not significant

To measure the total PrP^C^ amount in our fractionated samples, we integrated the intensity values from fraction 2 to 16 of NBH-inoculated samples as total PrP^C^, and expressed the integrated values for the same fractions from RML-infected animals as a percentage of this negative control reference value (Figs. 7 and 8). Interestingly, we detected a decrease in PrP^C^ protein levels at pre-clinical stages of the disease; this trend began at 90 DPI, and by 120 DPI, there was an approximately 29% reduction in PrP^C^ levels, which then further decreased at 150 DPI to 38% (Fig. 8). The profile of this decrease and the absolute percentage values were notably similar to a study using different methodologies (velocity gradients and conformation-dependent immunoassay) [5]. We next investigated the presence of high-molecular weight species at all timepoints in RML-inoculated animals and detected the first signs of such assemblies at 60 DPI (equivalent to 40% of the disease incubation period) (Fig. 7c and Supplementary Fig. 1). The distribution of multimeric assemblies detected at 60 DPI is significantly different from either NBH-inoculated or RML-inoculated animals at 30 DPI (*p* value ≤ 0.01), but is similar to that of terminal-stage RML mice (albeit with lower signal intensity); the columns corresponding to R_H_ values of 5–10 nm are slightly higher than that of NBH-inoculated animals, and there is generally a rise in signal for species with R_H_ values above 10 nm, with the highest peaks at 10–20 nm and 40–70 nm. This observation can be reconciled with our data from PK-digestion of total brain homogenates and indicate the presence of PK-resistant PrP^Sc^ at this timepoint (Fig. 6b).

### Polymeric Assemblies of PrP Have Distinct Protease Resistance Characteristics

**Fig. 9:**
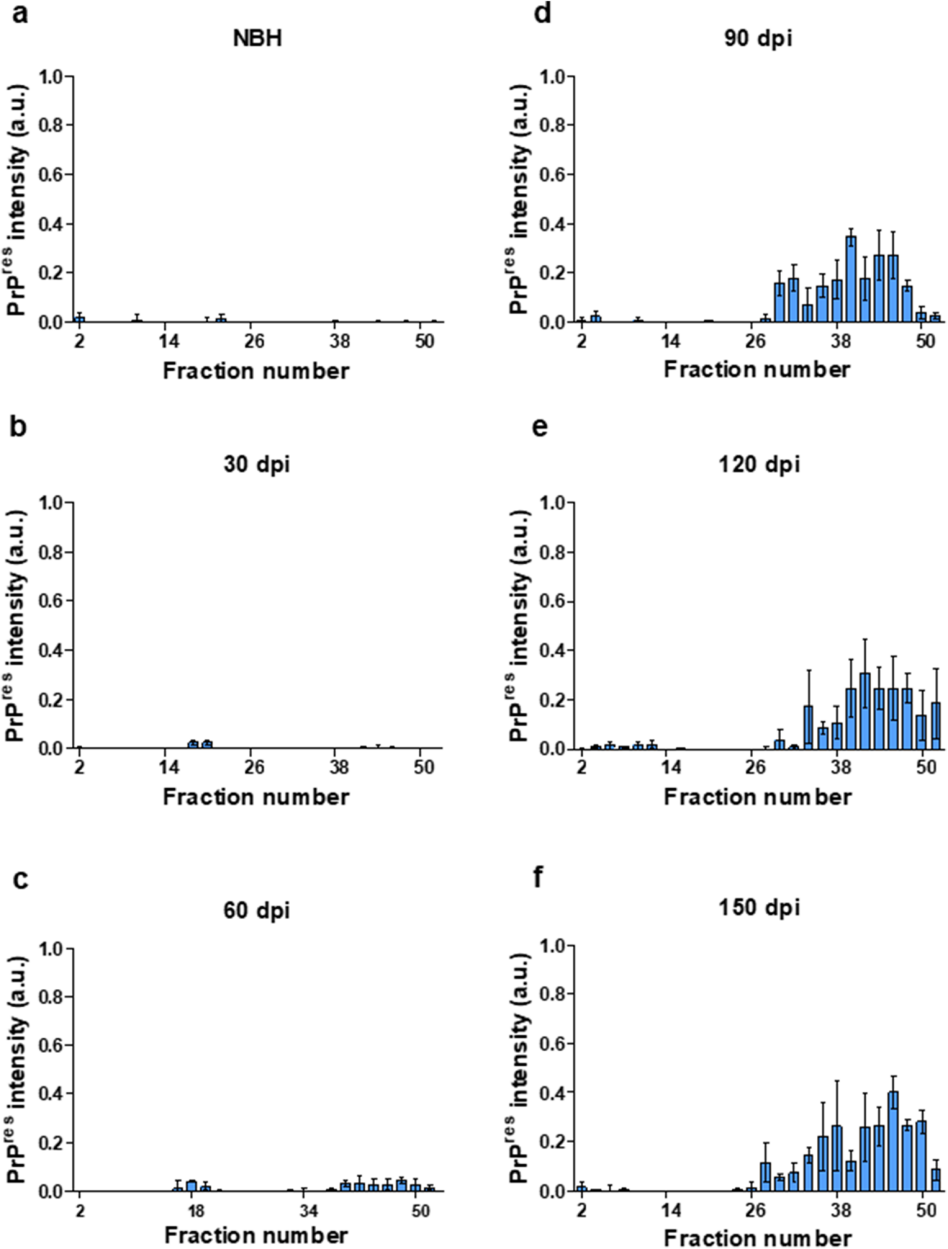
PK-resistant PrP^Sc^ in fractionated brain of RML-inoculated animals at different timepoints. The *Y*-axis represents the intensity of Pk-digested PrP in arbitrary units (see supplementary Fig. 2). **a** A representative graph from PK-digested fractionated brain of a NBH-inoculated animal at 150 DPI, **b** PK-digested fractionated brain of RML-inoculated mice sacrificed at 30 DPI, **c** PK-digested fractionated brain of RML-inoculated mice sacrificed at 60 DPI, **d** PK-digested fractionated brain of RML-inoculated mice sacrificed at 90 DPI, **e** PK-digested fractionated brain of RML-inoculated mice sacrificed at 120 DPI, and **f** PK-digested fractionated brain of RML-inoculated mice sacrificed at terminal stage (150 DPI). Biological replicates are as per Fig. 7. Error bars represent SD

Our initial assessment of PK-resistant PrP^Sc^ performed on supernatants from a 20,000×*g* sedimentation of solubilized brain homogenate revealed PK-resistant PrP^Sc^ material from 60 DPI onwards (Fig. 6b). To probe the biochemical characteristics of these assemblies, we investigated the PK-resistance of soluble brain homogenates subsequent to their fractionation under the mild denaturing conditions of AF4 fractionation, i.e., using dodecyl-β-D-maltoside and sarkosyl detergents but lacking exposure to thermal denaturation or use of chaotropic agents [40–42]. Interestingly, we observed that consistently, assemblies within specific fractions could have PK-resistant characteristics (Fig. 9 and Supplementary Fig. 2). A comparison of total brain homogenate and PK-digested AF4 fractions suggests that PK-resistant PrP^Sc^ assemblies appear at fraction #28 (corresponding to assemblies of c.a. 15 nm R_H_ and predicted to be composed of ~ 100 PrP monomers) and no PK-resistant PrP could be detected at earlier fractions.

**Fig. 10:**
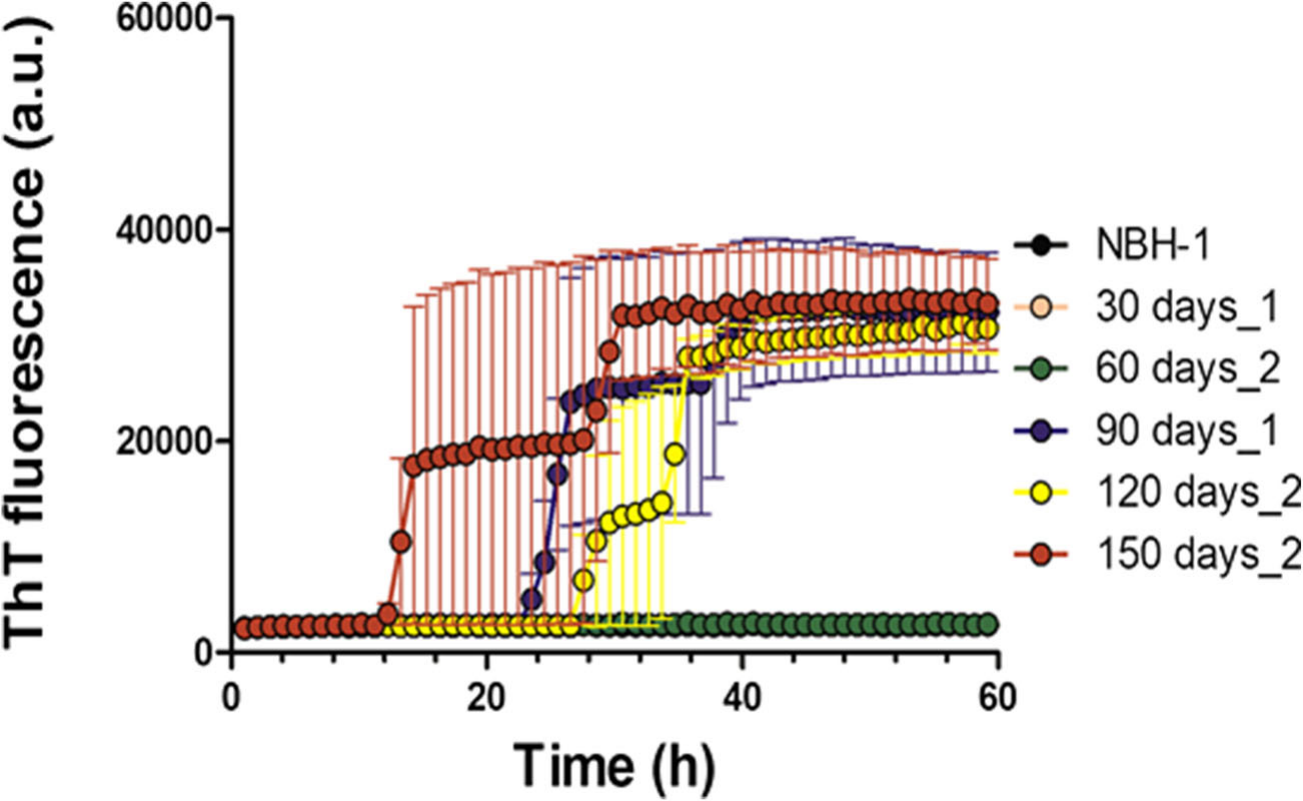
Seeding activity of total brain homogenate of RML-inoculated animals at different timepoints. Two microliters of 10% brain homogenate was used to assess the seeding potential of PrP species present in each timepoint. Data represents an average of 5 repeats. Error bars represent SEM

Next, to characterize the seeding potential of PrP^Sc^ assemblies from different timepoints, we assessed the seeding capacity of solubilized brain homogenate from different timepoints by RT-QuIC. Brain homogenates of RML-inoculated animals from 90 DPI, 120 DPI, and 150 DPI timepoints showed seeding activity in this assay (Fig. 10). Conversely, the seeding potential of PrP^Sc^ species at 60 DPI fell below the threshold of this assay configuration; from dilution series performed on 150 DPI brain homogenates, this threshold for positivity lies 100x below the amount present at this last timepoint. A similar distinction between 60 and 90 DPI timepoints was noted using a standard scrapie cell assay to titrate prion infectivity in a previous study (as discussed below; [5, 6]).

## Discussion

### Downregulation of the Cellular Prion Protein Precedes Neuronal Loss

In prion diseases, there is a plateau effect in the sub-clinical phase during which the infectivity titer, as well as the PrP^Sc^ levels, remains constant [4, 42]. This effect can be observed in our fractionation assay as well, where the net amount of high-molecular weight, PK-resistant assemblies of PrP is relatively constant at 90–150 DPI timepoints (with only 5% increase in PK-resistant PrP^Sc^ signal) (Fig. 9d–f, Supplementary Fig. 2). An early and important hypothesis in the field proposed that this extended incubation period is due to a limited number of replication sites in the host, which, with the benefit of hindsight can be equated with levels of the obligatory substrate for the production of PrP^Sc^, namely PrP^C^. Subsequently, data from native velocity gradient fractionation and conformation-dependent immunoassays defined a progressive fall in levels of PrP^C^ and the PrP-like, GPI-anchored shadoo protein (Sho) at pre-symptomatic stages of prion diseases; these data offer a simple explanation for the plateau in titer levels during the disease incubation period [5, 6, 43].

Our results using AF4 fractionation indicate that PrP^C^ protein levels trend downwards starting at 90 DPI and reached a significant decrease by 120 DPI (− 29% at 120 DPI and − 38% at 150 DPI). The profile of these data is strikingly similar to data obtained from different cohorts of RML-infected animals analyzed by entirely different methods (velocity gradient sedimentation and conformation-dependent immunoassay). Moreover, the absolute numerical values for each timepoint (− 24% at 120 DPI and − 37% at 150 DPI; [5]) are also strikingly similar, speaking to robustness. Since we observed no significant decrease in the number of neuronal cells until the last timepoint in our experiment (150 DPI), and while neuronal loss could be partially responsible for dropped PrP^C^ levels at this terminal stage, we conclude that falling PrP^C^ levels in the pre-clinical phase of disease are not necessarily due to cell loss and may require an alternative explanation. These data align with previous observations suggesting that this phase of disease features a controlled depletion of the substrate (PrP^C^), as well as the PrP-like shadoo protein (but not of other control proteins) and perhaps reflects a protective physiological response [5, 6, 44, 45]. Within the pre-clinical phase, changes in neuro-inflammatory markers were amongst the earliest changes noted in the brain (Fig. 3 and Fig. 5a, b). Based on our observations, inflammation preceded other events such as synaptic damage. The notion that non cell-autonomous events that involve activation of the innate immune system might relate to PrP^C^ downregulation may be worthy of investigation.

### Molecular Transition of Prion Protein and Formation of Prion Assemblies Is Initiated at Early Stages of the Disease

Despite the early recognition that PK-resistant PrP^Sc^ molecules correlate strongly with infectious titer, growing evidence points at the role of PK-sensitive PrP^Sc^ assemblies formed at preliminary stages of the disease in triggering pathogenesis. In conjunction with a cross-sectional experimental design, we took advantage of asymmetric flow field-flow fractionation to study molecular transitions of PrP. Unlike routine gradient centrifugation methods, AF4 has some advantages in speed, reproducibility, and higher resolution [46, 47]. In addition, AF4 can separate a wide range of particles within a complex sample (from a few nanometers to several micrometers) in the same run [48]. As employed here, an in-line DLS detector allows a precise measurement of the size of the eluting particles. The combination of these points makes AF4 particularly useful for studying aggregated PrP species [33, 49–51].

In data here PK-resistant PrP^Sc^ species appear at 90 DPI in immunohistochemistry assays, western blot analysis of solubilized brain homogenate first detects PK-resistant material at 60 DPI. This seeming discrepancy might reflect differences in sensitivity and detection limits of the two techniques [32]. Fractionation experiments also identify medium to high molecular weight assemblies of PrP starting from 60 DPI. These assemblies consist of mostly PK-sensitive species, but there are some PK-resistant species detected in fractions that must be composed of higher molecular weight particles. However, (RT-QuIC) analyses failed to document that PrP^Sc^ species at 30 and 60 DPI timepoints possessed seeding activity at 10^-3^ dilution, which was the highest input concentration used in our assay. While interference effects on assay read-outs cannot be excluded, these data align with a report where purified PK-resistant PrP^Sc^ from inoculated animals sampled at different timepoints using the same RML agent (and using a PK concentration of 20 μg/ml) were subjected to quantitative RT-QuIC and wherein material from 30 and 60 DPI did not manifest seeding activity [47]. Furthermore, broadly compatible results with 30 DPI not always above baseline, 45 and 60 DPI often above baseline, and 90 DPI always above baseline were obtained using the standard scrapie cell assay (SSCA; [52]) to measure infectious activity propagated within susceptible PK1 or L929 cells. Considered in toto, these different types of studies perhaps mitigate against trivial explanations of sensitivity thresholds and indicate, instead, that there qualitative, intrinsic changes in the nature of PrP conformers at the disease midpoint. Indeed, it has been proposed that prion assemblies at early stages of disease consist of two populations with distinct quaternary structures [13]. In the current study, a comparison of PK-digested fractionated brain homogenates from all timepoints indicated that in our model, polymers of 100-mer PrP or larger are the ones that manifest PK-resistance. The fact that smaller assemblies of PrP (R_H_ less than 15 nm) do not show PK-resistance at any stage during disease progression (even at terminal stage where there is high abundance of PrP assemblies with different size and R_H_ values) suggests that the relative PK-sensitivity of assemblies that elute in fractions less than 15 nm is not a function of abundance, but is indeed due to the intrinsic structural characteristics of the aforementioned assemblies.

Because of our study design, we cannot yet extrapolate to the question of region-specific differences in pathogenic PrP conformers. One previous study demonstrated similar seeding activity in a number of brain regions despite dissimilar aggregated prion particles and pathological distinctions [32]. In another study, region-specific homeostatic transcriptome signatures were replaced by region-independent neuroinflammation signature at clinical stages of the disease [53], although different regions did present unique transcriptome patterns at early timepoints. Extending the methods employed here to region-specific characterizations may have considerable merit.

## Conclusion

Analyses here confirm that, in accordance with our previous observations, PrP^C^ is downregulated at pre-clinical stages of prion disease [5, 6] and we suggest this decrement is not a trivial consequence of neuronal loss. This effect was detected in an approach without recourse to microdissection, perhaps speaking to robustness and the participation of potentially important cell biological events. It is eminently plausible that a superseding, adjusted approach considering discrete neuroanatomical regions would define neuronal loss, or signs of inflammation at yet earlier timepoints.

Considering PrP conformers, comparative analyses amongst undigested brain homogenate fractions and PK-digested ones suggest that despite the substantial increase in the levels of PK-resistant PrP^Sc^, PK-digestion at any timepoint during disease progression reveals a specific subpopulation of assemblies with similar biophysical characteristics. Our data suggest that PK-sensitive oligomers of PrP^Sc^ that appear as early as 40% of the disease incubation period are associated with triggering disease-related events such as neuroinflammation and gross pathological changes in the brain, as well as perhaps triggering failed or incomplete neuroprotective responses that can only be perceived by their molecular signatures.

Finally, the observations reported here are restricted to the RML isolate of mouse-adapted prions. Further work on deciphering the correlation between molecular transition of PrP and disease pathogenesis in different strains with distinct courses of pathogenesis and in tandem with region-specific analyses could provide useful information towards understanding the molecular mechanisms of strain-specific characteristics observed amongst prion isolates.

## Electronic supplementary material

Supplementary Fig. 1 The immunoblots of total PrP in AF4 fractions corresponding to all timepoints. Twenty six fractions obtained from AF4 fractionation of brain samples (every second fraction between 1-52) were subjected to immunoblotting, with the data representing two biological replicates for each sample group. Since the protein concentration in all fractions were below detection limits of protein concentration assays (BCA assay kit), equal volume of each fraction (10 μl) was loaded on gel. Antibody Sha31(1/5000) (PNG 4948 kb)

High Resolution Image (TIF 366 kb)

Supplementary Fig. 2 The immunoblots of PK-resistant PrP fractions obtained from corresponding to all timepoints. Fractions as per supplementary Fig. 1 were incubated with 20 μg/mL of PK in the presence of 20 μg bovine serum albumin (BSA) as the sacrificial substrate to account for the low mass of PrP in eluted fractions, and to assure constant total protein mass in all samples. The reaction was stopped after 1 hour and samples were subjected to immunoblotting. Antibody Sha31(1/5000) (PNG 4948 kb)

High Resolution Image (TIF 317 kb)
