# Supplementary figures and images for: Quaternary Structure Changes for PrPSc Predate PrPC Downregulation and Neuronal Death During Progression of Experimental Scrapie Disease
Source: Mol Neurobiol. 2020 Sep 21;58(1):375–90. doi: 10.1007/s12035-020-02112-z (PMC7695655; doi:10.1007/s12035-020-02112-z)

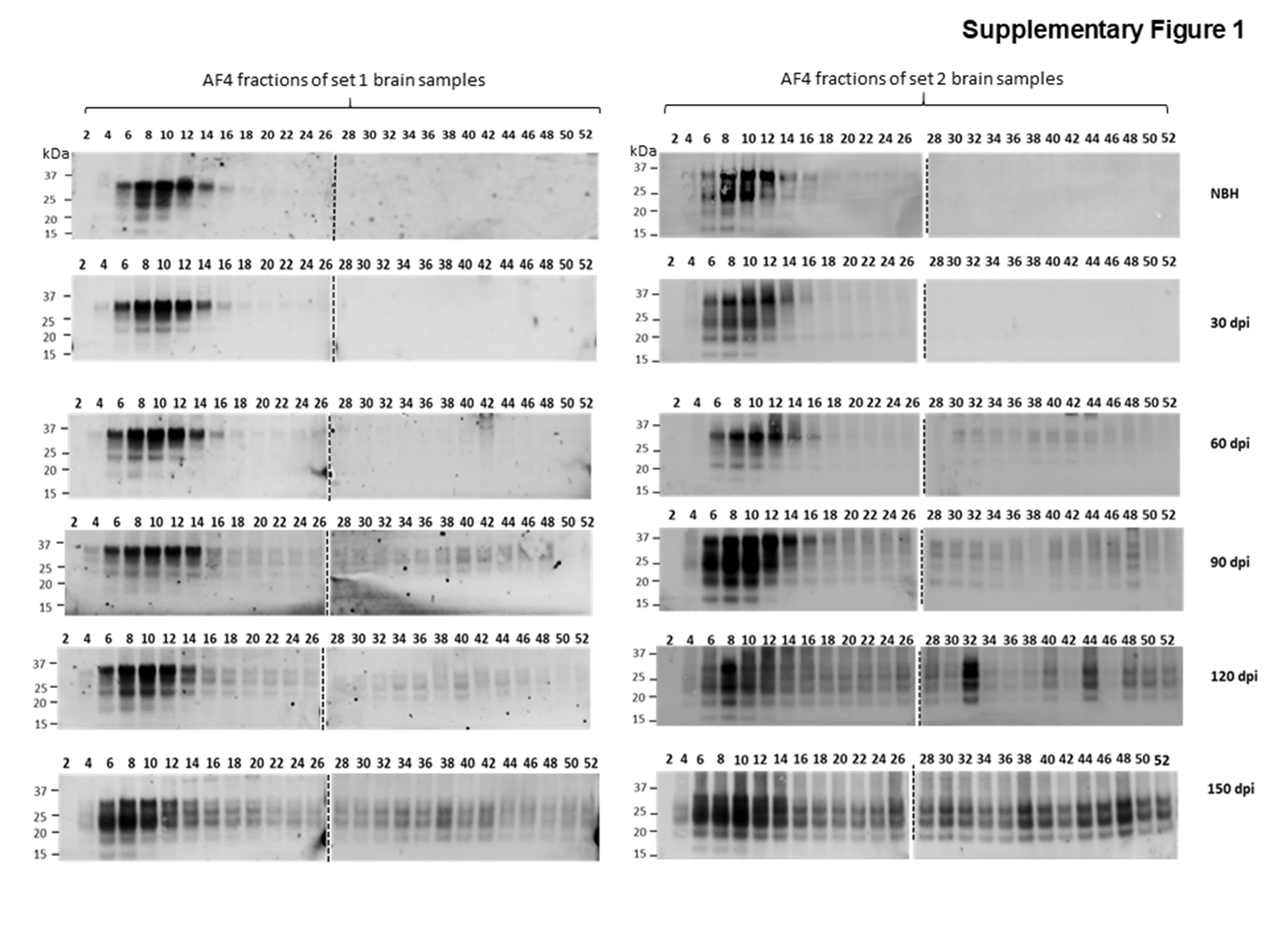

Supplement: Supplementary file 1 — The immunoblots of total PrP in AF4 fractions corresponding to all timepoints. Twenty six fractions obtained from AF4 fractionation of brain samples (every second fraction between 1-52) were subjected to immunoblotting, with the data representing two biological replicates for each sample group. Since the protein concentration in all fractions were below detection limits of protein concentration assays (BCA assay kit), equal volume of each fraction (10 μl) was loaded on gel. Antibody Sha31(1/5000) (PNG 4948 kb) [file 12035_2020_2112_Fig11_ESM.png]

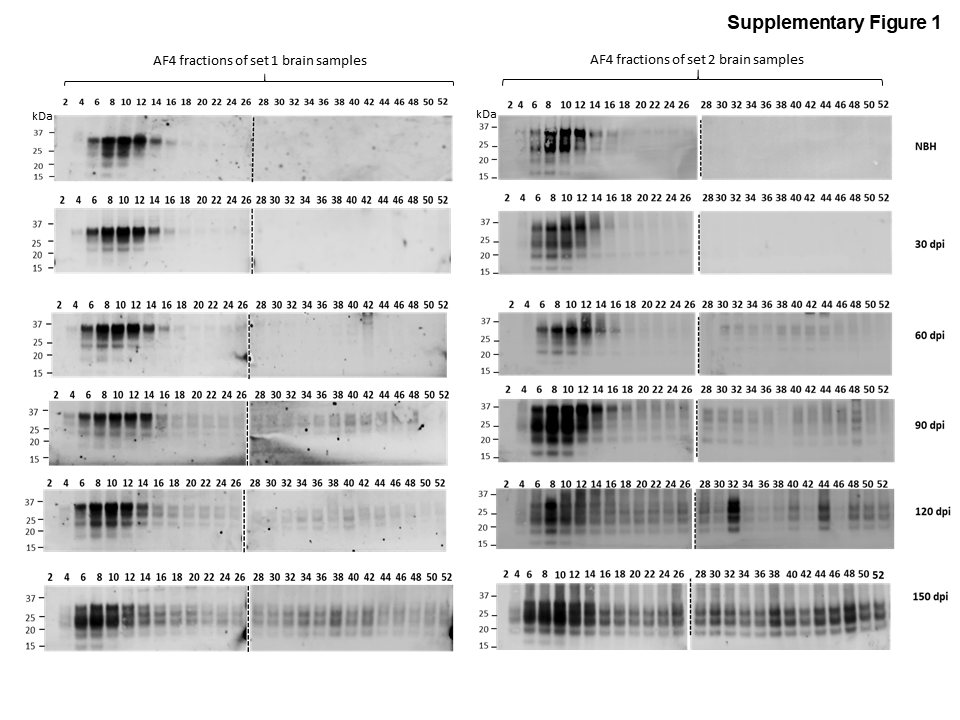

Supplement: Supplementary file 2 — High Resolution Image (TIF 366 kb) [file 12035_2020_2112_MOESM1_ESM.tif]

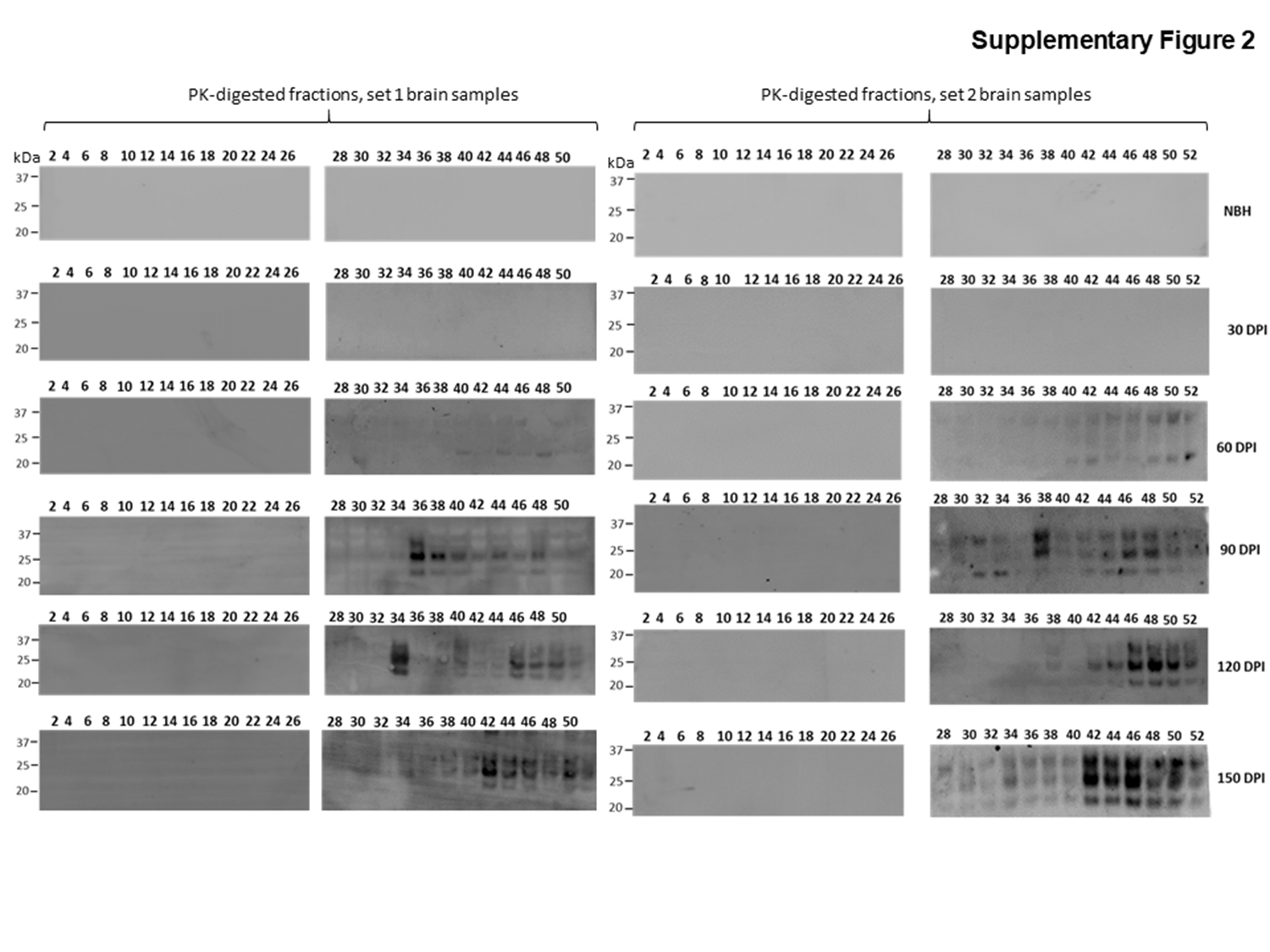

Supplement: Supplementary file 3 — The immunoblots of PK-resistant PrP fractions obtained from corresponding to all timepoints. Fractions as per supplementary Fig. 1 were incubated with 20 μg/mL of PK in the presence of 20 μg bovine serum albumin (BSA) as the sacrificial substrate to account for the low mass of PrP in eluted fractions, and to assure constant total protein mass in all samples. The reaction was stopped after 1 hour and samples were subjected to immunoblotting. Antibody Sha31(1/5000) (PNG 4948 kb) [file 12035_2020_2112_Fig12_ESM.png]

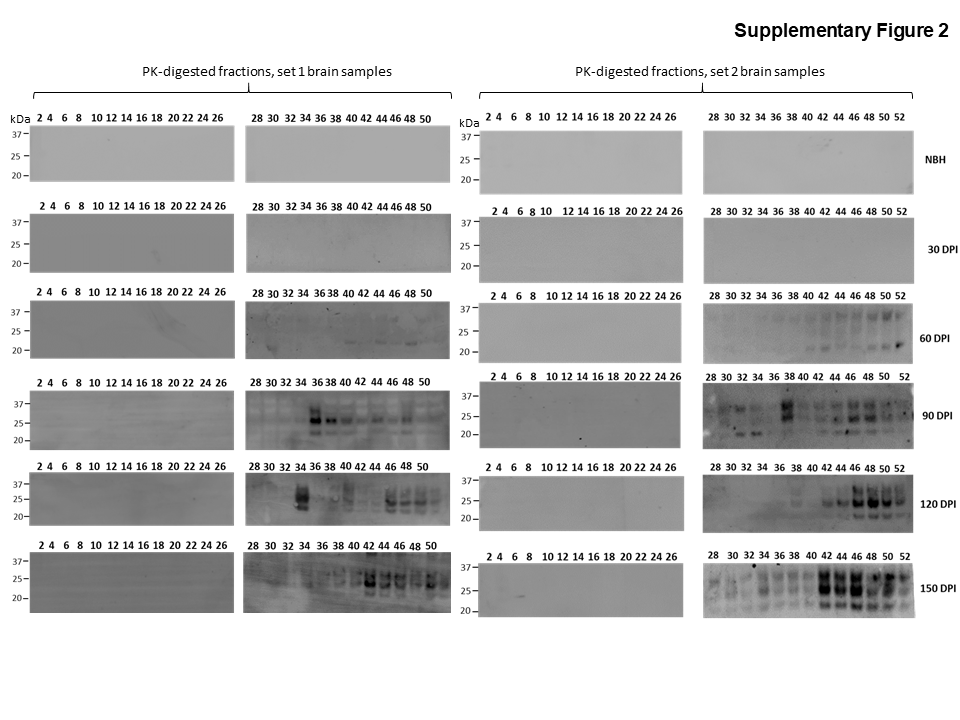

Supplement: Supplementary file 4 — High Resolution Image (TIF 317 kb) [file 12035_2020_2112_MOESM2_ESM.tif]
